# Anterior segment configuration as a predictive factor for refractive outcome after cataract surgery in patients with glaucoma

**DOI:** 10.1186/s12886-016-0359-1

**Published:** 2016-10-18

**Authors:** Young Cheong Kim, Mi Sun Sung, Hwan Heo, Sang Woo Park

**Affiliations:** 1 Department of Medical Science Graduate School, Chonnam National University, Gwangju, Republic of Korea; 2 Department of Ophthalmology and Research Institute of Medical Sciences, Chonnam National University Medical School and Hospital, 42 Jebong-ro, Dong-gu, Gwangju, 61469 Republic of Korea; 3 Center for Creative Biomedical Scientists, Chonnam National University, Gwangju, Republic of Korea

**Keywords:** Refractive outcome, Cataract surgery, Anterior segment configuration, Lens vault

## Abstract

**Background:**

To compare refractive outcomes after cataract surgery between patients with closed-angle and open-angle glaucoma and evaluate the influence of preoperative factors on refractive outcomes in patients with glaucoma.

**Methods:**

Patients diagnosed with glaucoma and who underwent uncomplicated cataract surgery were enrolled in this retrospective observational study. We collected data including age, history of prior laser peripheral iridotomy and trabeculectomy, type of glaucoma, manifest refraction, intraocular pressure, axial length, and various anterior segment parameters using anterior-segment optical coherence tomography. Factors associated with unsatisfactory refractive outcome at postoperative 6 month were evaluated.

**Results:**

A total of 143 eyes (143 subjects) were included. Of these, 49 and 94 had closed-angle and open-angle glaucoma, respectively. At postoperative-6 month evaluation, the mean absolute error (MAE) predicted by the SRK-II and SRK-T formulae was 0.67 ± 0.61 and 0.81 ± 0.66 diopters (D), respectively. The overall predictability of achieving within ± 1.0 D of target was 76.92 % and 72.73 %, respectively. At a cutoff value of 1.0 D for MAE, there was no statistical significant difference in refractive outcome between the closed-angle and open-angle glaucoma groups. Logistic regression modeling showed that large lens vault (LV) was a significant predictor of unsatisfactory refractive outcome after cataract surgery in patients with glaucoma.

**Conclusions:**

When considering cataract surgery in patients with glaucoma, surgeons should recognize that the refractive outcomes may be unsatisfactory in eyes with large LV.

## Background

The most critical element of a successful cataract surgery is obtaining excellent postoperative refractive outcomes. Although recent advancements in ocular biometry measurement, surgical techniques, intraocular lens (IOL) manufacturing and IOL power formula have improved the predictability of IOL power calculations, unsatisfactory refractive outcomes are still a concern following cataract surgery. Since the prevalence of cataract and glaucoma increases with age, the management of cataract in glaucoma patients is a common clinical challenge. Additionally, the accurate calculation of IOL power and identification of possible factors affecting this predictability are crucial to ensure desired postoperative results.

Previous studies suggested that several preoperative factors including short or long axial length, keratometric values, corneal asphericity, pupil size, and anterior chamber depth were associated with the unpredictability of refractive outcomes [1–6]. Specifically, eyes with angle closure tend to have a myopic or hyperopic shift in the postoperative refractive outcome after cataract surgery [7–10]. The anatomic characteristics of eyes with angle closure, such as large lens capsules, posterior shifting of the capsular bag, a decrease in axial length due to lowered intraocular pressure (IOP) after cataract surgery, and zonular weakness, might explain these unsatisfactory refractive outcomes [8, 11, 12]. Most studies of refractive outcomes in patients with glaucoma have been conducted in patients with closed-angle glaucoma; few reports are published on refractive outcomes in patients with open-angle glaucoma.

Recently, the introduction of anterior-segment optical coherence tomography (AS-OCT) has enabled us to perform quantitative measurements of biometric parameters, such as anterior chamber depth (ACD), iris curvature, angle width, and lens position [13–16]. In addition, several studies reported that anterior segment parameters measured by AS-OCT may have clinical significance in differentiating the type of angle closure, assessing the effect of laser peripheral iridotomy (LPI), and predicting IOP lowering after cataract surgery [17–21]. Hence, in the present study, we hypothesized that baseline anterior segment configuration might be associated with refractive outcomes after cataract surgery. The aim of present study was to investigate the relative importance of preoperative parameters, including anterior segment configuration (measured using AS-OCT), with respect to refractive outcomes in patients with glaucoma.

## Methods

### Subjects

This is a retrospective observational study. We performed a review of medical records for patients diagnosed with glaucoma and who underwent uncomplicated phacoemulsification with posterior chamber IOL implantation by a single surgeon (S.W.P) in Chonnam National University Medical School and Hospital between January 2013 and October 2014. The study protocol adhered to the tenets of the Declaration of Helsinki and was approved by the Institutional Review Board of Chonnam National University Hospital.

The following inclusion criteria were used: i) age > 40 years; ii) in the bag fixation of the IOL; iii) 1-piece acrylic IOLs must have been used (SN60WF; Alcon Laboratories, Fort Worth, TX, USA); and iv) a minimum follow-up period of 6 months. Exclusion criteria included: i) secondary angle closure due to an intumescent lens, uveitis, ocular trauma, choroidal effusion, and medication (e.g., topiramate); ii) previous ocular surgeries other than prior LPI or trabeculectomy; iii) intraoperative complications, including anterior or posterior capsular tears; iv) combined procedures such as phacoemulsification/trabeculectomy; v) postoperative macular edema; vi) additional surgery within 6 months postoperatively; and vii) invalid biometry, lack of visual acuity, IOP, and refractive data at postoperative 6 months visits. If both eyes of a single patient underwent cataract operations, only the first operated eye was selected for these analyses.

### Patient assessment

The data collected included: i) age at phacoemulsification, ii) history of prior LPI and trabeculectomy, iii) type of glaucoma (closed-angle or open-angle), and iv) history of acute angle closure. Closed-angle glaucoma and open-angle glaucoma patients were categorized based on recent diagnostic classifications of glaucoma [22]. In brief, closed-angle glaucoma is defined as an eye with an occludable angle (greater than 270° of posterior trabecular meshwork that cannot be seen with a Posner 4-mirror gonioprism in the primary position without indentation), evidence of angle dysfunction [elevated IOP (>21 mmHg), appositional or synechial contact between the peripheral iris and posterior trabecular meshwork, and excessive pigment deposition on the trabecular surface], and glaucomatous optic neuropathy such as optic nerve head excavation or thinning of the neuroretinal rim and corresponding visual field defects. Eyes with a history of angle-closure attack were also included in the closed-angle group. Open-angle glaucoma was defined as an eye with an open angle confirmed by gonioscopy, IOP of greater than 21 mmHg on diurnal testing with Goldmann applanation tonometry (GAT) before treatment, glaucomatous optic neuropathy, and corresponding visual field defects. Eyes with normal tension glaucoma (an IOP of less than 21 mmHg on diurnal testing with GAT before treatment) were also included in the open-angle group.

Patients underwent a comprehensive ophthalmic examination preoperatively and postoperatively, including: i) measurement of best corrected visual acuity (BCVA), ii) manifest refraction, and iii) IOP using a GAT. BCVA was converted to logarithm of the minimum angle of resolution (logMAR) and manifest refraction was converted into spherical equivalent (SE) for analysis. The following measurements were also made preoperatively: i) axial length and lens thickness using the Lenstar LS900 (Haag-Streit, Bern, Switzerland), ii) keratometry using an automated keratometer (KR8900; Topcon Corp, Tokyo, Japan), and iii) other anterior segment parameters using AS-OCT. Considering the possibility that effects of too large values and too small values may offset each other in evaluating the association between axial length and refractive outcome, we additionally investigated the association between deviation from mean axial length and refractive outcome. Deviation from mean axial length was defined as the absolute values of difference between the individual axial length and mean axial length. Deviation from mean ACD was also calculated.

### Surgical procedures

Surgical procedures were performed under topical or retrobulbar anesthesia. In all cases, a temporal 2.8-mm clear corneal incision, continuous curvilinear capsulorrhexis, hydrodissection, and phacoemulsification with the Infiniti Vision system (Alcon Laboratories) were performed to remove the cataract. A foldable 1-piece acrylic IOL was implanted in the capsular bag and following the operation, the surgeon confirmed that the IOL was accurately implanted. All study subjects used Acrysof SN60WF (Alcon Laboratories) IOLs with manufacturer-recommended A-constants of 118.7. The IOL power was biometrically calculated using the SRK-II and SRK-T formulae.

### Measurements of anterior segment parameters

Images of the anterior segment were obtained using a commercially available AS-OCT device (Visante OCT; Carl Zeiss Meditec, Dublin, CA, USA). After directing the subjects to look straight at an internal fixation target within the device in a sitting position, one experienced technician who was blinded to other clinical findings obtained the image in a dark room. The scans were centered on the pupil and horizontal cross-sectional images of the nasal and temporal angle (0–180°) were obtained until the quality were sufficient to analyze. Since assessment of the superior and inferior angles often requires manual manipulation of the eyelids, which may distort the angle, we did not evaluate the images from vertical scans. All subjects underwent AS-OCT imaging under identical conditions. Imaging in subjects with a history of primary angle closure was performed after the resolution of an acute episode. A single examiner (S.W.P) selected the best images with no motion artifacts, good visibility of the scleral spur, and no image artifacts from the eyelids.

**Fig. 1:**
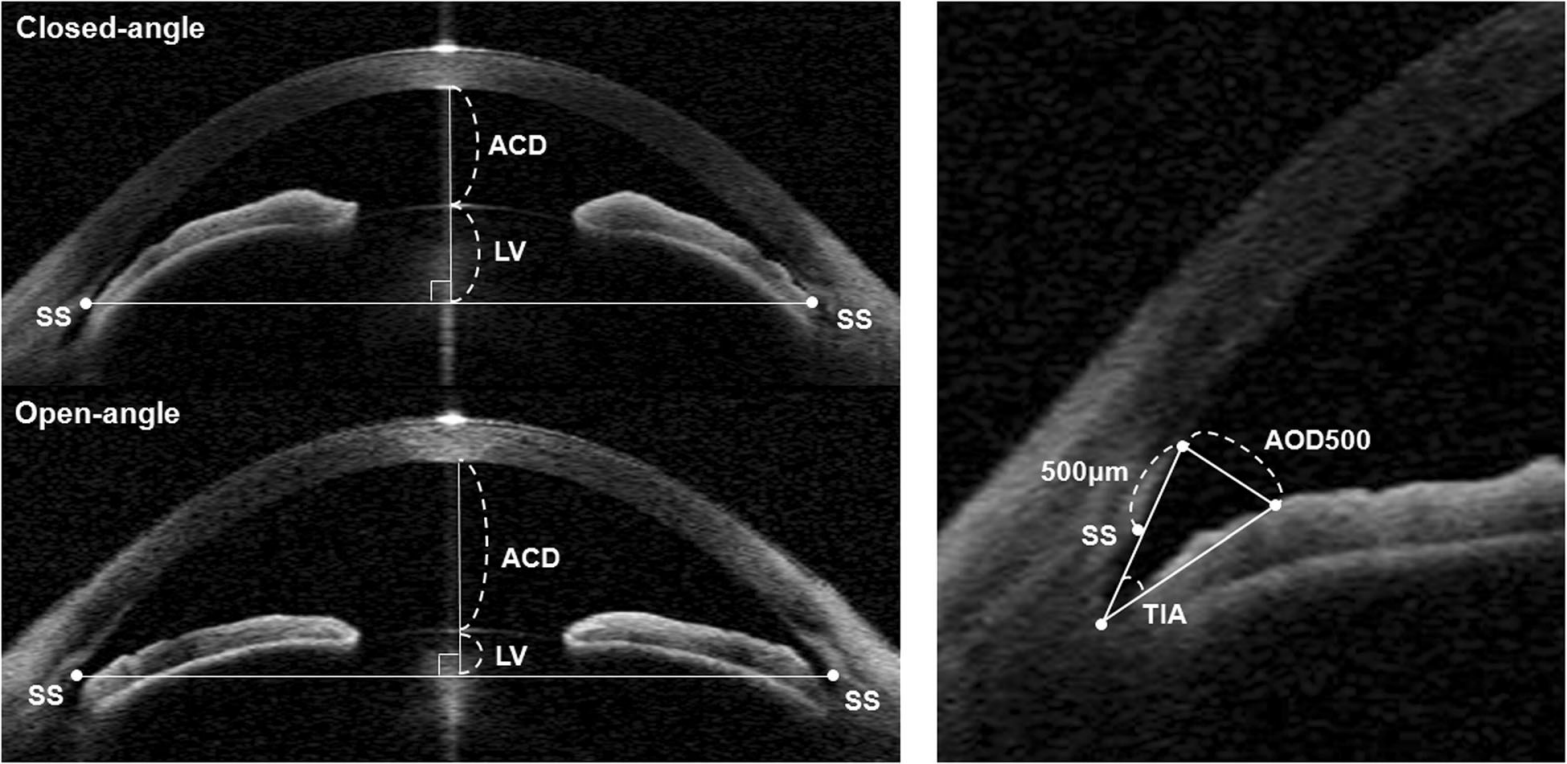
Anterior-segment optical coherence tomography image illustrating measurements of anterior chamber depth (ACD), lens vault (LV), angle opening distance at 500 μm (AOD500), and trabecular iris angle (TIA). Points SS indicate the sclera spur

Two independent examiners (Y.C.K and M.S.S) who were blinded to other clinical information analyzed images using custom software (Iridocorneal module, Carl Zeiss Meditec). We measured the following 4 parameters: i) ACD, defined as the distance from the corneal endothelium to the anterior lens surface; ii) lens vault (LV), defined as the maximum perpendicular distance between the anterior pole of the crystalline lens and the horizontal line connecting the two scleral spurs; iii) angle opening distance at 500 μm (AOD500), defined as the perpendicular distance from the trabecular meshwork at 500 μm anterior to the scleral spur to the anterior iris surface; and iv) trabecular iris angle (TIA), defined as an angle measured with the most angle recess point and the arms of the angle passing through a point on the trabecular meshwork 500 μm from the scleral spur and the point on the iris perpendicularly opposite (Fig. 1). Among the 4 parameters, AOD500 and TIA were obtained both nasally and temporally and the means of the measured values in each position were calculated.

### Outcome measures

Based on the postoperative 6 month evaluations, SE was compared with the expected refractive outcomes as determined by the SRK-II and SRK-T formulas. The mean absolute error (MAE) is defined as the absolute difference between the intended formula-derived SE refractive target and the actual postoperative SE. Based on the MAE value, subjects was divided into two groups: those with satisfactory refractive outcome (MAE < 1.0 diopters [D]) and those with unsatisfactory refractive outcome (MAE ≥ 1.0 D).

### Statistical analysis

Statistical analysis was performed using a commercially available statistical software package (SPSS, ver. 18.0; SPSS Inc, Chicago, Illinois, USA). Interobserver reproducibility was evaluated by calculating the intraclass correlation coefficient (ICC), which ranges from 0 to 1; higher values indicating higher correlation of the units and better repeatability. Bland and Altman reported that an ICC of 0.8 to 1.0 indicates high reliability [23]. The normality of distribution was verified using the Shapiro-Wilk normality test. Baseline characteristics were reported in counts and proportions or mean ± standard deviation (SD) values as appropriate. Groups were compared using the Chi-square test or Fisher’s exact test for categorical variables and independent *t* test for continuous variables. If we have small than 5 subjects in any cell of data table, Fisher’s exact test was used. Comparisons between preoperative and postoperative IOP in each group were made using the paired *t* test.

Logistic regression analysis was used to evaluate baseline factors including parameters of anterior segment configuration associated with unsatisfactory refractive outcome after uncomplicated cataract surgery. First, each variable was analyzed in a univariate model. Next, all variables with a significance level of less than 0.10 were included in the multivariate model. The role of each variable is expressed in odds ratio (OR) and its 95 % confidence intervals (CIs). *P* values less than 0.05 were considered statistically significant.

## Results

All 143 eyes of 143 subjects met the inclusion criteria for enrollment in the present study. Of the 143 eyes, 49 were classified as having closed-angle glaucoma and 94 were classified as having open-angle glaucoma. Baseline characteristics of the subjects are presented in Table 1. The mean age was 66.40 ± 10.64 years; 60 subjects were male and 83 were female. There was no significant difference in age, preoperative BCVA, proportion of subjects with prior trabeculectomy history, or lens thickness among the closed-angle and open-angle glaucoma groups. However, the closed-angle glaucoma group showed a significantly higher proportion of female patients (*P* = 0.019) and shorter axial length (*P* < 0.001) than the open-angle glaucoma group. All parameters of anterior segment configuration were also significantly different between the two groups (all *P* < 0.001) with excellent interobserver reproducibility (all ICCs > 0.9).

**Table 1:** Baseline characteristics of eyes with closed-angle and open-angle glaucoma

Variables	Total (*n* = 143)	Closed-angle glaucoma (*n* = 49)	Open-angle glaucoma (*n* = 94)	*P* value
Age at surgery (yrs)	66.40 ± 10.64	66.08 ± 9.23	66.56 ± 11.34	0.798^a^
Sex (male/female)	60/83	14/35	46/48	0.019^b^
Preoperative BCVA (logMAR)	0.65 ± 0.78	0.84 ± 0.97	0.54 ± 0.63	0.057^a^
Preoperative IOP (mmHg)	15.62 ± 4.96	17.10 ± 6.73	14.85 ± 3.52	0.032^a^
Prior LPI (yes/no)	19/124	19/30	0/94	<0.001^c^
Prior trabeculectomy (yes/no)	19/124	4/45	15/79	0.299^c^
Axial length (mm)	22.95 ± 1.12	22.33 ± 1.03	23.28 ± 1.03	<0.001^a^
Deviation from mean axial length (mm)	0.84 ± 0.74	0.89 ± 0.81	0.81 ± 0.71	0.157^a^
Mean keratometry (D)	44.01 ± 2.32	44.38 ± 1.61	43.82 ± 2.60	0.172^a^
Glaucoma diagnosis (NTG/POAG)	49/45	NA	49/45	NA
History of acute angle closure (yes/no)	36/107	36/13	NA	NA
Lens thickness (mm)	4.66 ± 0.69	4.85 ± 0.93	4.58 ± 0.54	0.191^a^
Parameters of anterior segment configuration				
ACD (mm)	2.30 ± 0.68	1.64 ± 0.48	2.64 ± 0.50	<0.001^a^
Deviation from mean ACD (mm)	0.56 ± 0.36	0.72 ± 0.38	0.50 ± 0.33	0.001^a^
LV (mm)	0.74 ± 0.44	1.18 ± 0.35	0.52 ± 0.29	<0.001^a^
AOD500 (μm)	319.07 ± 139.37	210.36 ± 60.09	375.74 ± 135.35	<0.001^a^
TIA (degree)	30.63 ± 9.70	24.61 ± 6.77	33.76 ± 9.54	<0.001^a^

*BCVA* best-corrected visual acuity, *logMAR* logarithm of the minimum angle of resolution, *IOP* intraocular pressure, *LPI* laser peripheral iridotomy, *D* diopter, *NTG* normal tension glaucoma, *POAG* primary open-angle glaucoma, *ACD* anterior chamber depth, *LV* lens vault, *AOD500* angle opening distance at 500 μm, *TIA* trabecular iris angle

^a^Independent *t* test for closed-angle and open-angle glaucoma

^b^Chi-squared test for closed-angle and open-angle glaucoma

^c^Fisher exact test for closed-angle and open-angle glaucoma

At postoperative 6 month evaluations, IOP decreased significantly in both groups (both *P* < 0.001). There was no difference in IOP change between the two groups (*P* = 0.276). MAE predicted by the SRK-II and SRK- T formulae was 0.67 ± 0.61 and 0.81 ± 0.66 D, respectively. The overall predictability of achieving within ± 1.0D of target by SRK II and SRK T was 76.92 and 72.73 %, respectively. Postoperative refractive errors predicted by both SRK II and SRK-T formula showed overcorrection, resulting in more myopic refractive errors than expected. Comparing the MAE value and percentage of eyes that achieved a postoperative SE within ± 1.0 D from the preoperative predicted refraction, the open-angle glaucoma group had a smaller MAE value and higher percentage of eyes with postoperative SE within ± 1.0 D from the preoperative predicted refraction than the closed-angle group. However, there was no statistical significant difference in refractive outcomes between the closed-angle and open-angle glaucoma groups (Table 2).

**Table 2:** Postoperative outcome after uncomplicated cataract surgery

Variables	Total (*n* = 143)	Closed-angle glaucoma (*n* = 49)	Open-angle glaucoma (*n* = 94)	*P* value
Postoperative BCVA (logMAR)	0.32 ± 0.71	0.45 ± 0.94	0.26 ± 0.56	0.184^a^
Postoperative IOP (mmHg)	14.59 ± 4.67	15.78 ± 6.40	13.98 ± 3.32	0.071^a^
IOP change (mmHg)	−1.03 ± 2.36	−1.33 ± 2.21	−0.87 ± 2.43	0.276^a^
Predicted SE (D)				
SRK II	−0.24 ± 0.64	−0.15 ± 0.67	−0.29 ± 0.62	0.219^a^
SRK T	0.03 ± 0.56	0.24 ± 0.52	−0.07 ± 0.58	0.002^a^
Postoperative SE (D)	−0.51 ± 0.95	−0.49 ± 1.06	−0.52 ± 0.89	0.830^a^
MAE (D)				
SRK II	0.67 ± 0.61	0.76 ± 0.55	0.62 ± 0.64	0.200^a^
SRK T	0.81 ± 0.66	0.94 ± 0.64	0.75 ± 0.66	0.091^a^
Deviation from predicted SE (within 1.00 D), *n* (%)				
SRK II	110 (76.92)	35 (71.43)	75 (79.79)	0.298^b^
SRK T	104 (72.73)	32 (65.31)	72 (76.60)	0.169^b^

*BCVA* best-corrected visual acuity, *logMAR* logarithm of the minimum angle of resolution, *IOP* intraocular pressure, *SE* spherical equivalent, *D* diopter, *MAE* mean absolute error

^a^Independent *t* test for closed-angle and open-angle glaucoma

^b^Chi-squared test for closed-angle and open-angle glaucoma

**Fig. 2:**
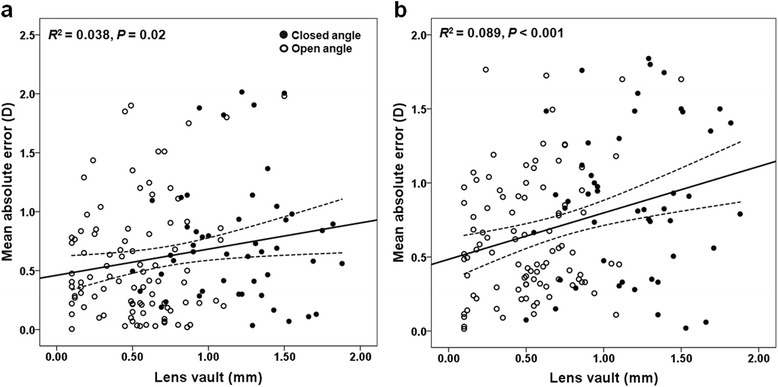
Scatter plots showing the relationship between mean absolute error versus lens vault in eyes with closed angle and open angle glaucoma. **a** mean absolute error predicted by SRK-II formulae, **b** mean absolute error predicted by SRK-T formulae. Closed-angle glaucoma indicated by solid circles and open-angle glaucoma indicated by open circles. The dashed lines are the 95 % confidence intervals for the solid trend lines

The result of comparisons between the eyes with satisfactory refractive outcome and unsatisfactory refractive outcomes are presented in Table 3. At a cutoff value of 1.0 D for MAE predicted by the SRK-II and SRK-T formula, 33 and 39 eyes were classified as the unsatisfactory outcome group, respectively. Among the 33 eyes with unsatisfactory refractive outcomes based on the SRK-II formulae, 25 showed myopic shift (closed-angle glaucoma group (*n* = 11); open-angle glaucoma group (*n* = 14)) and 8 showed hyperopic shift (closed-angle glaucoma group (*n* = 3); open-angle glaucoma group (*n* = 5)). Similarly, among the 39 eyes with unsatisfactory outcomes based on the SRK-T formulae, 32 showed myopic shift (closed-angle glaucoma group (*n* = 15); open-angle glaucoma group (*n* = 17)) and 7 showed hyperopic shift (closed-angle glaucoma group (*n* = 2); open-angle glaucoma group (*n* = 5)). In both SRK-II and SRK-T formulae derived IOL power calculation, LV was significantly different between the two groups (*P* = 0.036 and *P* = 0.017, respectively). The results of the logistic regression also showed that large LV was a significant predictor of unsatisfactory outcome after cataract surgery in patients with glaucoma (OR = 2.331 and 38.293; *P* = 0.039 and 0.020 using the SRK-II and SRK-T formulae, respectively) (Table 4). Figure 2 shows the statistically significant association between MAE and LV. The regression was greater in SRT-T (*P* < 0.001, *R* ^*2*^ = 0.089) than in SRK-II (*P* < 0.020, *R* ^*2*^ = 0.038).

**Table 3:** Comparisons of characteristics of the satisfactory and unsatisfactory refractive outcome group

Variables	SRK II			SRK T		
	Satisfactory outcome MAE < 1.0 D (*n* = 110)	Unsatisfactory outcome MAE ≥ 1.0 D (*n* = 33)	*P* value	Satisfactory outcome MAE < 1.0 D (*n* = 104)	Unsatisfactory outcome MAE ≥ 1.0 D (*n* = 39)	*P* value
Age at surgery (yrs)	66.59 ± 10.68	65.76 ± 10.62	0.694^a^	66.30 ± 11.08	66.67 ± 9.49	0.854^a^
Sex (male/female)	48/62	12/21	0.548^b^	47/57	13/26	0.254^b^
Preoperative BCVA (logMAR)	0.62 ± 0.72	0.75 ± 0.93	0.397^a^	0.58 ± 0.68	0.81 ± 0.98	0.185^a^
Postoperative BCVA (logMAR)	0.27 ± 0.65	0.51 ± 0.88	0.084^a^	0.23 ± 0.56	0.57 ± 0.98	0.052^a^
Preoperative IOP (mmHg)	15.31 ± 4.43	16.67 ± 6.38	0.260^a^	15.46 ± 4.52	16.05 ± 6.02	0.528^a^
Postoperative IOP (mmHg)	14.22 ± 4.12	15.85 ± 6.06	0.155^a^	14.40 ± 4.37	15.10 ± 5.43	0.427^a^
IOP change (mmHg)	−1.09 ± 2.29	−0.82 ± 2.60	0.562^a^	−1.06 ± 2.42	−0.95 ± 2.21	0.807^a^
Prior LPI (yes/no)	12/98	7/26	0.126^b^	12/92	7/32	0.406^b^
Prior trabeculectomy (yes/no)	13/97	6/27	0.383^b^	11/93	8/31	0.164^b^
Axial length (mm)	22.97 ± 1.06	22.88 ± 1.31	0.668^a^	23.07 ± 1.11	22.63 ± 1.10	0.035^a^
Deviation from mean axial length (mm)	0.81 ± 0.68	0.92 ± 0.92	0.447	0.84 ± 0.73	0.82 ± 0.79	0.850^a^
Mean keratometry (D)	44.04 ± 2.42	43.90 ± 1.98	0.750	43.91 ± 2.42	44.29 ± 2.03	0.384^a^
Glaucoma diagnosis (closed/open)	35/75	14/19	0.298^b^	32/72	17/22	0.169^b^
History of acute angle closure (yes/no)	24/86	12/21	0.111	25/79	11/28	0.667^b^
Lens thickness (mm)	4.75 ± 0.54	4.27 ± 1.08	0.199^a^	4.74 ± 0.55	4.31 ± 1.07	0.247^a^
Anterior segment configuration parameters						
ACD (mm)	2.33 ± 0.65	2.17 ± 0.77	0.228^a^	2.35 ± 0.65	2.16 ± 0.75	0.134^a^
Deviation from mean ACD (mm)	0.55 ± 0.34	0.65 ± 0.42	0.192	0.55 ± 0.35	0.63 ± 0.41	0.252^a^
LV (mm)	0.71 ± 0.43	0.87 ± 0.46	0.036^a^	0.69 ± 0.43	0.89 ± 0.45	0.017^a^
AOD500 (μm)	321.39 ± 135.70	311.36 ± 152.93	0.718^a^	321.68 ± 134.31	312.11 ± 153.67	0.716^a^
TIA (degree)	30.79 ± 9.45	31.05 ± 12.81	0.715^a^	30.62 ± 9.40	30.64 ± 10.59	0.993^a^

*BCVA* best-corrected visual acuity, *logMAR* logarithm of the minimum angle of resolution, *IOP* intraocular pressure, *LPI* laser peripheral iridotomy, *D* diopter, *NTG* normal tension glaucoma, *POAG* primary open-angle glaucoma, *ACD* anterior chamber depth, LV lens vault, *AOD500* angle opening distance at 500 μm, *TIA* trabecular iris angle

^a^Independent *t* test

^b^Chi-squared test

**Table 4:** Factors associated with unstable refractive outcome after uncomplicated cataract surgery in glaucomatous eyes

Variables	SRK II		SRK T			
	Univariate analysis		Univariate analysis		Multivariate analysis^a^	
	Odds ratio (95 % CI)	*P* value	Odds ratio (95 % CI)	*P* value	Odds ratio (95 % CI)	*P* value
Age at surgery (for each year older)	0.993 (0.958–1.029)	0.692	1.003 (0.969–1.039)	0.853		
Female gender	1.355 (0.607–3.025)	0.459	1.649 (0.764–3.560)	0.203		
Preoperative BCVA (for each logMAR increase)	1.226 (0.766–1.960)	0.396	1.419 (0.911–2.211)	0.121		
Preoperative IOP (mmHg)	1.051 (0.978–1.130)	0.179	1.023 (0.953–1.099)	0.527		
IOP change (mmHg)	1.050 (0.892–1.236)	0.559	1.020 (0.873–1.192)	0.805		
Prior LPI	2.423 (0.856–6.865)	0.116	1.394 (0.484–4.014)	0.538		
Prior trabeculectomy	2.009 (0.728–5.547)	0.178	2.182 (0.805–5.915)	0.125		
Axial length (mm)	0.925 (0.650–1.316)	0.665	0.681 (0.474–0.979)	0.038	0.931 (0.348–2.491)	0.887
Deviation from mean axial length (mm)	1.216 (0.736–2.010)	0.445	0.952 (0.576–1.575)	0.849		
Mean keratometry (D)	0.974 (0.831–1.143)	0.749	1.091 (0.897–1.327)	0.383		
Closed-angle glaucoma	1.579 (0.711–3.509)	0.262	1.739 (0.815–3.709)	0.152		
History of acute angle closure	2.048 (0.883–4.749)	0.112	1.241 (0.541–2.847)	0.610		
Lens thickness (mm)	0.388 (0.138–1.109)	0.132	0.435 (0.161–1.175)	0.098	0.119 (0.014–1.015)	0.060
Parameters of anterior segment configuration						
ACD (mm)	0.701 (0.394–1.248)	0.227	0.658 (0.380–1.139)	0.135		
Deviation from mean ACD (mm)	2.008 (0.703–5.731)	0.193	1.795 (0.661–4.875)	0.251		
LV (mm)	2.331 (1.274–5.578)	0.039	2.713 (1.171–6.285)	0.020	38.293 (1.766–830.314)	0.020
AOD500 (μm)	0.999 (0.997–1.002)	0.719	0.999 (0.997–1.002)	0.714		
TIA (degree)	0.992 (0.953–1.034)	0.713	1.000 (0.963–1.039)	0.992		

*CI* confidencial interval, *BCVA* best-corrected visual acuity, *logMAR* logarithm of the minimum angle of resolution, *IOP* intraocular pressure, *SE* spherical equivalent, *D* diopter, *LPI* laser peripheral iridotomy, *NTG* normal tension glaucoma, *POAG* primary open-angle glaucoma, *ACD* anterior chamber depth, *LV* lens vault, *AOD500* angle opening distance at 500 μm, *TIA* trabecular iris angle

^a^Only variables with a *P* value of less than .10 in the univariate analysis were included in the multivariate model

## Discussion

Predicting refractive outcomes depends on exact preoperative biometric measurements such as axial length and keratometry, and the IOL power calculation formula used. However, even using newer-generation IOL power calculation formulas, the prediction of postoperative IOL position in the eye is not perfect in usual clinical setting. For cataract surgery in patients with glaucoma, postoperative refractive outcomes have been an issue of concern, especially in patients with closed-angle glaucoma. Kang et al. [8] and Joo et al. [24] compared results between angle-closure glaucoma and a control group with open angle and reported that IOL power calculation was less accurate in angle-closure glaucoma patients. Rhiu et al. [25] showed a similar result in eyes that underwent three-piece IOL implantation and suggested that short axial length and shallow anterior chamber may be the main causes responsible for unsatisfactory refractive outcomes.

These studies suggested that cataract surgery in eyes with shallow anterior chambers may result in considerable changes of the anterior chamber configuration and it would be difficult to estimate the postoperative IOL position accurately. However, in our previous study focused on factors affecting refractive outcomes in patients with acute primary angle closure, we found no associations between the refractive outcomes and ocular biometry including preoperative axial length and ACD [10]. In addition, little has been studied about refractive outcomes in patients with open-angle glaucoma. Hence, in the present study, we quantify the various parameters from anterior segment configuration using AS-OCT and evaluate the association of such parameters with refractive outcomes in patients with closed-angle and open angle glaucoma. This was a novel study investigating the role of anterior segment configuration as a predictive factor for refractive outcome after cataract surgery in patients with glaucoma. In our study, the percentage of eyes with unsatisfactory refractive outcome (MAE ≥ 1.0 D) was about 25 % and the greater LV becomes, the more the MAE value increases.

LV, which represents the volume of the lens anterior to the plan of the scleral spur, has been reported as the parameter associated with angle closure [26]. Nongpiur et al. [27] suggested that in eyes with symptomatic angle closure and a large LV, cataract surgery can be considered even in the presence of good visual acuity, because LV was not associated with increasing myopic refractive error and decreasing visual acuity. How et al. [28] reported that LPI did not result in a significant change of LV, although anterior chamber angle increased significantly after LPI. Theoretically, increase in LV implies the more anterior position of lens in the eye. This change can be attributed to the thickened lens or zonular laxity with age. We may speculate that eyes with large LV predispose individuals to larger displacements of IOL position, resulting in unsatisfactory refractive outcomes after cataract surgery. In our study, LV was significantly associated with refractive outcomes in patients with glaucoma.

From previous studies, we may consider several factors affecting refractive outcomes after cataract surgery in patients with glaucoma. One of these factors is a change of IOP, which can cause lengthening or shortening of globes, leading to inaccurate axial length measurements. It has been known that phacoemulsification leads to angle widening and IOP reduction [29, 30]. Francis et al. [31] found a correlation between the IOP change and axial length change. In a study evaluating the effect of prior trabeculectomy on refractive outcome, Zhang et al. [4] also demonstrated that cataract surgery in patients with prior trabeculectomy had significantly greater refractive surprise than those in the control groups. However, in the present study, there was no significant difference in preoperative IOP, postoperative IOP and the amount of IOP change between the satisfactory and unsatisfactory refractive outcome groups. Similarly, there was no association between the history of prior trabeculectomy and refractive outcomes in our study.

It was reported that SRK-II and SRK-T formulae could be inaccurate in predicting the postoperative refraction after cataract surgery in eyes with short or long axial length and shallow ACD. However, we could not find the statistically significant association between the axial length and ACD and refractive outcome. Regarding the axial length, the result can be probably explained by the distribution axial length of our study subjects. Since the number of subjects with short axial length (≤21.5 mm) and long axial length (≥25 mm) were only 16 (11.2 %) patients (short axial length 10, long axial length 6), the parameters associated with axial length would not significantly affect the refractive outcome in this study. Previously, Aristodemou et al. [32] evaluated the accuracy of SRK T formula in the various axial length groups and found that SRK-T formula showed similar refractive outcome for eyes with axial length between 21.5 and 25.0 mm. In terms of the ACD, our result indicates that the anterior chamber depth alone were not enough to predict the postoperative refractive outcome and other factors such as LV should also be taken into account.

Another factor affecting refractive outcomes is cataract density. Ueda et al. [33] previously reported that MAE was significantly correlated with cataract density. Because of insufficient medical record, we could not analyze the effect of cataract density on refractive outcomes. However, during phacoemulsification, no significant difference was recorded in cumulative dissipated energy between groups (data not shown), and therefore, the effect of cataract density might be not significant.

The present study has some limitations that need to be considered. First, a larger sample size study is required to confirm our data. Additionally, because all patients were Asian, it is unclear whether similar associations would be seen in other racial groups. Second, we did not have postoperative AS-OCT measurements. Evaluation of changes in anterior segment configuration after cataract surgery and association of such changes with refractive outcomes might have been useful to further characterize our findings. Third, there are many other factors that may affect refractive outcomes after cataract surgery. However, due to the retrospective nature of the study, we could not collect enough information from medical records to quantify the impact of these other factors.

## Conclusions

In summary, our results highlight the importance of anterior segment configuration in predicting refractive outcomes after cataract surgery. We suggest that surgeons consider preoperative LV when planning cataract surgery in patients with glaucoma.

## Abbreviations

ACD: Anterior chamber depth
AOD500: Angle opening distance at 500 μm
AS-OCT: Anterior-segment optical coherence tomography
BCVA: Best corrected visual acuity
CIs: Confidence intervals
D: Diopters
GAT: Goldmann applanation tonometry
ICC: Intraclass correlation coefficient
IOL: Intraocular lens
IOP: Intraocular pressure
logMAR: Logarithm of the minimum angle of resolution
LPI: Laser peripheral iridotomy
LV: Lens vault
MAE: Mean absolute error
OR: Odds ratio
SD: Standard deviation
SE: Spherical equivalent
TIA: Trabecular iris angle
